# Evaluation of a Workplace Health and Wellbeing Training Course Delivered Online and Face-To-Face

**DOI:** 10.3390/ijerph15112422

**Published:** 2018-10-31

**Authors:** Zenobia Talati, Emily Davey, Carly Grapes, Trevor Shilton, Simone Pettigrew

**Affiliations:** 1 School of Psychology, Curtin University, Kent St, Bentley, WA 6102, Australia; simone.pettigrew@curtin.edu.au; 2 National Heart Foundation, Perth, WA 6008, Australia; emily.davey@health.wa.gov.au (E.D.); trevor.shilton@heartfoundation.org.au (T.S.); 3 Cancer Council Western Australia, Perth, WA 6008, Australia; carly.grapes@belmont.wa.gov.au

**Keywords:** online training, face-to-face training, workplace champions, workplace health, wellbeing

## Abstract

Organisations may benefit from training champions to promote healthy workplace environments and initiatives. This study compared the perceived usefulness and relative effectiveness of an employee training course offered via online and face-to-face formats. Individuals who took part in the training course were assessed on their perceived competence and confidence to implement changes pre- and post-training. Repeated measures Analysis of Variance (ANOVA) and a *t*-test were conducted to test for significant differences between pre- and post-training scores and/or mode of training, respectively. Although the face-to-face training course was rated as slightly more useful, there were no significant differences between the two modes of training for the other dependent variables, and both modes led to significantly greater perceived competence and confidence post-training. These findings demonstrate the potential benefits of training employees to implement changes in their workplaces.

## 1. Introduction

Workplaces are an important context for promoting health and wellbeing [1]. They have a substantial coverage of the population in terms of the number of people in paid employment and the number of hours spent in the workplace [2]. Workplace environments may be improved through the provision of healthier facilities (e.g., showers and change rooms to encourage active transport to and from work, adequate food preparation and storage facilities to encourage healthier foods to be brought from home, sit-to-stand workstations), policies (e.g., a healthy catering policy), and participation and education initiatives (e.g., sponsoring sporting teams, healthy eating education sessions). These improvements can facilitate and normalise healthy behaviours when individuals do not have the awareness, ability, and/or motivation to engage in these behaviours of their own accord [3]. For example, workplace policies and interventions targeting smoking, nutrition, alcohol consumption, and physical activity (known as SNAP behaviours) have been found to result in improvements in related behaviours and outcomes [4,5,6].

There are, however, a number of barriers to the successful implementation of workplace health and wellbeing initiatives. Not all workplaces have adequate resources to adopt comprehensive programs, and among those that do, obtaining buy-in from managers and employees to accept and/or participate in such programs can be difficult [7]. One way to assist in overcoming these barriers is through a ‘ground up’ approach in which organisations empower change agents within the workplace (e.g., occupational health and safety officers, human resource officers, supervisors, or other workplace health champions) to improve health and wellbeing in their workplaces. Champions can be equipped with the skills and knowledge required to implement sustainable healthy initiatives in their workplace to better support employee health and wellbeing. Training champions to promote workplace interventions (health-related or otherwise) is promoted within the organisational health literature but is understudied [8]. Two recent trials reported positive outcomes from training champions to implement strategies to reduce the time employees spent sitting at work and increase their physical activity [9,10]. In focus group discussions from one study [10], champions described how they encouraged other employees to engage in physical activity. Many reported using both direct encouragement (e.g., through in-person conversations and regular emails) and indirect encouragement (e.g., by setting up a social network to facilitate physical activity within the organization). Other strategies included disseminating key program messages to their peers, remaining enthusiastic and persistent, providing encouragement without judgement, and role modelling desirable behaviours [10]. In the other study [9], champions were instructed to use similar direct and indirect encouragement strategies, as well as creating an action plan and celebrating successes. A three-month evaluation of the program revealed that it reduced self-reported workplace sitting time by approximately 30 min per day. However, little is known about the effectiveness of training champions to improve workplace health across multiple risk factors such as those relating to SNAP behaviours. Thus, one aim of this study was to determine whether providing training to workplace champions could increase workplace capacity to better support employee health and wellbeing.

Employees can obtain the knowledge and skills required for implementing workplace health and wellbeing interventions through online or face-to-face training channels. Advantages to online training include lower cost, greater convenience, and wider reach (especially to those in rural and remote geographical areas) [11]. Advantages of face-to-face training include greater engagement with content and ease of including group interaction elements [12]. Most of the research comparing online to face-to-face training has been conducted on students in educational contexts [13]. This work has typically found few (if any) differences in key learning outcomes such as knowledge and skills. The limited past research specifically on workplace health education programs has found small positive effects of both online [14] and face-to-face [15] modes of training. For example, one study [14] comparing an online training program, designed to improve diet, reduce stress, and increase physical activity, to a control group (receiving print materials) found that the online training program produced a significantly greater increase in positive attitudes towards healthy eating but no significant change in other outcome variables. Similarly, a meta-analysis of face-to-face training interventions found only small changes in diet and physical activity compared to their respective control groups [15]. However, these studies focused only on physical activity or nutrition programs and did not directly compare courses with equivalent content that are delivered through different modes. A second aim of this study was thus to assess whether mode of training (online vs. face-to-face) impacts on the effectiveness of workplace training programs with comparable content.

## 2. Materials and Methods

### 2.1. Participants

The training courses were promoted through social media (e.g., through Facebook and LinkedIn using paid and unpaid posts), Healthier Workplace Western Australia (HWWA) newsletters, the HWWA website, and other relevant association newsletters. Data were collected between June 2016 and April 2018, during which time 222 individuals completed online training and 182 individuals attended face-to-face training. In total, 168 individuals completed the pre and post surveys for the online training (response rate of 76%) and 174 completed the pre and post surveys for face-to-face training (response rate of 96%). Participants were a mix of managers (16%), individuals working in human resources/occupational health and safety (57%), employees with a general interest in health and wellbeing (20%), and other/not reported roles (6%). Out of all the participants taking part in the online and face-to-face training, 40% came from large organisations with over 200 employees. The remaining participants came from medium organisations with 20–199 employees (33%), small organisations with less than 20 employees (17%), or did not report their organization size (10%). This study was approved by a University Human Research Ethics Committee (Approval code: RDHS-39-16, date of approval: 23 August 2017). Before completing the surveys, respondents provided consent for their data to be used for research purposes.

### 2.2. Training Course

This study compared the relative effectiveness of a training course delivered using online and face-to-face modes in terms of the outcome variables of perceived course usefulness and perceived confidence and ability to make changes. The aim of the training was to help employees identify and implement best-practice strategies to improve health and wellbeing in their workplaces (described in more detail below). The online training is completed in four self-paced modules (expected to take roughly 20 min each). The face-to-face training runs for approximately three hours and includes group discussions and activities. While both courses cover the same conceptual content, the face-to-face course includes time for group discussion, tailored feedback, and breaks. The training is offered as one component of the Healthier Workplace WA (HWWA) program, which is a comprehensive program freely available to workplaces within Western Australia. The program (funded by the Health Department of Western Australia and administered through the National Heart Foundation Western Australia Division and Cancer Council Western Australia) intends to build the capacity of organisations to implement workplace health and wellbeing initiatives. This objective is achieved via the provision of free support, advice, training, tools, and resources to implement workplace health and wellbeing initiatives.

HWWA training covers the following topics: (1) SNAP risk factors for chronic disease; (2) the importance of workplace health promotion; and (3) how to plan for, implement, and evaluate changes made in the workplace. After completing the training, individuals are expected to be able to:

- Describe why the workplace is a priority setting for health promotion
- Identify the costs of an unhealthy workplace
- Identify the benefits of investing in a workplace health program
- List the key modifiable lifestyle behaviours that influence employee health
- Describe the national guidelines relevant to physical activity, healthy eating, alcohol, and smoking
- List best-practice workplace health promotion strategies
- Identify the different stages of change employees may be at in relation to a healthy lifestyle
- Identify areas of risk and suitable intervention strategies relevant to their workplace

### 2.3. Training Evaluation

Before commencing and immediately after taking part in the training, participants were asked to complete a survey. On a 5-point scale, respondents were asked to indicate the degree to which they could “identify and implement best practice strategies to target” healthy eating, physical activity, smoking, and alcohol in their workplace. The scores on these four items were aggregated to reflect their ability to identify SNAP risk factors. Respondents also answered the following items on a 5-point scale:

- I understand the steps involved in creating a workplace health and wellbeing action plan (with 1 being ‘Strongly disagree’ and 5 being ‘Strongly agree’).
- I can develop a business case to gain (further) support from management to implement healthy lifestyle initiatives in my workplace (with 1 being ‘Strongly disagree’ and 5 being ‘Strongly agree’).
- How confident are you that you can make changes in your workplace to promote healthy lifestyle behaviours to staff (with 1 being ‘Not very confident’ and 5 being ‘Very confident’)?
- How useful was the training you completed (with 1 being ‘Not at all useful’ and 5 being ‘Very useful’)?

Aside from the usefulness measure, all items were measured both pre- and post-training to enable assessment of improvement.

### 2.4. Analysis

Repeated measures Analysis of Variance (ANOVA) was conducted to test for a significant difference between the online and face-to-face training and pre- and post-training scores. A *t*-test was used to assess for differences in perceived usefulness according to mode of training.

## 3. Results

As shown in Table 1, all the dependent variables assessed increased significantly from pre- to post-training participation. There were no significant differences between the two modes of training for any of the dependent variables (*p* > 0.10), with the exception of the face-to-face training being rated slightly more useful than the online training.

## 4. Discussion

This study looked at the effectiveness of training designed to inform employees about strategies to improve health and wellbeing in their workplace and empower them to make changes. The relative effectiveness of two modes of training (online and face-to-face) were compared. Effectiveness was measured through perceived ability to identify SNAP risk factors, ability to create a health and wellbeing action plan, ability to create a business case for improving workplace health and wellbeing, confidence to make changes in the workplace, and perceived usefulness of the training. Both modes of training resulted in significant pre to post increases in perceived competence and confidence to implement changes. Between the different modes of training, only ratings of usefulness differed, with the face-to-face training being rated slightly but significantly higher than online training.

A limitation of this study was that people were not randomised to online or face-to-face training, and instead they chose the format that was most suitable for them. It is thus possible that individual attributes influenced the form of training attended and/or the resulting outcomes. In addition, the very wide geographical dispersion of the state of Western Australia meant that all face-to-face courses were conducted in the metropolitan area and those working in regional areas would have been limited to selecting the online format of the course. This may have introduced a systematic bias to the results. However, given that no significant differences were found between the two modes of training in this real-world study, the results obtained from the non-randomised design are useful in indicating that these two modes of training can be equally effective. The outcome measures used in this study focused on perceived capacity to make changes. Further work is needed to explore whether a self-reported increase in capacity among champions translates into changes being made in the workplace. It is also important to note that while this study only evaluated the effect of mode of delivery, there are other factors related to the person and organizational environment (e.g., self-efficacy, support from management) that are likely to influence the effectiveness of the training.

## 5. Conclusions

These findings provide further evidence to inform a sparse literature about the potential benefits of training workplace change agents and champions to implement health and wellbeing initiatives in their workplaces. Specifically, this study offers positive insights into the role of training courses delivered using either an online or face-to-face format. Given the low relative cost in conducting online training and its ability to reach remote workplaces, it was encouraging to note that there were few significant differences in effectiveness between the modes for the dependent variables. Results indicate that both modes of training can influence employees’ perceived confidence and ability to identify risks and to make strategic changes to their workplace to enhance the health and wellbeing of the workforce.

## Figures and Tables

**Table 1:** Pre and/or post training scores for online and face-to-face training modes of training among Western Australian employees engaging in the training program between June 2016 and April 2018.

Dependent Variables	Mean (Online)		Mean (Face-To-Face)		p
	Pre	Post	Pre	Post	
Perceived ability to identify SNAP risk factors ^a^	15.53	17.49	14.77	17.42	<0.001
Perceived ability to create a health and wellbeing action plan ^a^	3.59	4.48	3.41	4.55	0.03
Perceived ability to create a business case for improving workplace health and wellbeing ^a^	3.74	4.31	3.60	4.25	<0.001
Confidence to make changes in the workplace ^a^	3.84	4.21	3.66	4.20	<0.001
Perceived usefulness ^b^	-	4.42	-	4.66	<0.001

SNAP = smoking, nutrition, alcohol, and physical activity. Note: all items measured on a 5 point scale. ‘Perceived ability to identify SNAP risk factors’ is the aggregate of four items, and thus scores range from 5 to 20. ^a^ Significant differences in pre to post scores across dependent variables assessed with repeated measures Analysis of Variance. ^b^ Significant differences in perceived usefulness of training modes assessed with independent samples *t*-test.
